# The Uses of Hydrogen Peroxide

**Published:** 1908-04-04

**Authors:** Dan M'Kenzie


### SPECIAL ARTICLE.

### THE USES OF HYDROGEN PEROXIDE.

By DAN M'KENZIE, M.D.(Glasg.), C.M.

There seems to be a kind of natural law directing the manner in which new remedies rise into favour. Some come rapidly to the front; some, again, are brought to notice stamped with the imprimatur of honest workers, and backed by experiments which fully explain the action of the new agent. As examples of the second class we may mention nitrite of amyl, thyroid-gland substance, and adrenalin—remedies which have fully realised the high hopes raised by their behaviour in the laboratory. This class comes quickly into favour, is everywhere received and trusted, and remains popular for almost any length of time. A third group there is, the members of which slip in, as it were, by a back-door, and have to wait patiently, sometimes for many years, before they finally obtain recognition.

Hydrogen peroxide is a member of the third group. Introduced as long ago as the pre-antiseptic days, it fell into desuetude, or, to speak more correctly, was scarcely used at all, on account of its unreliability, instability, and acid impurities. The experience of recent years and the possibility of obtaining the solution free from impurities have, however, altered our opinion of the remedy, and we now look upon it as an agent invaluable for its purifying and hæmostatic qualities, not yet recognised by the general body of the profession as fully as its merits warrant.

### Hæmostatic Properties.

As a hæmostatic, hydrogen peroxide is most useful in controlling that form of hæmorrhage known as "parenchymatous," when every little arteriole and capillary is copiously pouring out blood. Under these circumstances the ten-volume solution of the liquid applied by means of swabs will dry up the bleeding surface with almost magical rapidity. This action reminds one of the hæmostatic action of hot water, but the manner in which the two substances act is different. Hot water stimulates the muscular tissues of the vessels to contract, while hydrogen peroxide induces hæmostasis by causing a rapid formation of fibrin in the mouths of the severed vascular channels. Hydrogen peroxide is, of course, easier to handle than hot water, and it is free from the risk of burning the parts.

While $H_2O_2$ may be used as a hæmostatic under almost any circumstances, it is specially relied upon in bone operations like the radical mastoid, when free oozing from the osseous wound prevents the all-important continual inspection of the depths of the cavity. It is also of the greatest service in the surgery of the nose, where operations are liable to be accompanied and followed by an amount of hæmorrhage not only inconvenient, but even at times dangerous. In epistaxis also, which, it will be remembered, is usually due to the rupture of a small vessel low down in the anterior part of the nasal septum, hydrogen peroxide applied to the bleeding area on a tampon will suffice in most cases to bring the hæmorrhage to a standstill. Of other varieties of hæmorrhage which have been successfully combated by this remedy we may mention uterine hæmorrhage, in which it has been found that $H_2O_2$ solution injected into the interior of the uterus, or applied on strips of gauze, is sufficient to lead to a cessation of the bleeding. On the other hand, too much should not be expected of it. For example, it is highly improbable that internal hæmorrhage, from the stomach or bowels for instance, can be controlled by peroxide of hydrogen, because the solution must undergo decomposition long before it reaches a bleeding-spot so remote.

### Antiseptic and Deodorant Properties.

However useful hydrogen peroxide has proved to be as a hæmostatic, there can be no doubt that its prime service consists of its antiseptic, deodorant, and purifying qualities. It is true that the bacteriologists have denied to it the power of destroying bacterial life, but it is also true that we are acquainted with a number of substances which enjoy the confidence of the surgeon, in spite of the fact that bacteriologists refuse to credit them with being bactericides. Iodoform is the most familiar of these agents, despised and rejected in the laboratory, but welcomed at the bedside. Hydrogen peroxide is another, for, when applied to foul discharging wounds and ulcers, it does undoubtedly act as a cleanser and as an antiseptic in the widest sense. And, moreover, it is no exaggeration to say that in this respect hydrogen peroxide is the equal, if not the superior, of many powerful chemical antiseptics.

In the presence of blood, pus, or necrotic tissues, hydrogen peroxide undergoes decomposition, parting with an atom of its oxygen—a chemical change which gives rise to a free and rapid effervescence of minute bubbles. These bubbles, rising from the depths of a wound to the surface, bear with them minute particles of dead tissue, pus, and débris generally, just as tiny bubbles of gas in effervescing soda-water seize on minute pieces of cork and hurry them to the surface. Hydrogen peroxide owes its cleansing and antiseptic qualities solely to this mechanical action, say the bacteriologists, for they are unwilling to grant to the nascent oxygen any considerable property of killing bacteria. Whatever the explanation may be, does not really matter very much. About the practical result there is no dispute.

### Illustration of Uses.

In the cleansing of all sorts of septic wounds, ulcers, and cavities, hydrogen peroxide is unrivalled. In suppuration of the middle ear, for instance, it may be used with wonderful benefit as "drops," the ten-volume solution being diluted with an equal quantity of water. After the characteristic foaming is over the ear is carefully dried out and suitable astringent or chemically antiseptic solutions instilled. It should not be used indiscriminately, for we must remember that where any suspicion exists of the possibility of mastoid infection, where, for example, the drainage of the middle ear through the tympanic membrane is imperfect, and where, therefore, we fear that the effervescing liquid will not find a free exit into the meatus, then hydrogen peroxide is better avoided.

In all varieties of "septic" and membranous sore-throat the solution is of great value. The writer has seen a septic exudate on the tonsil vanish entirely a few hours after using it. The best way to apply the remedy in these cases is to spray the solution accurately on to the inflamed surface. Gargling is a very imperfect and unreliable method, and mopping with swabs is unpleasant and often painful. In like manner septic conditions about the mouth, catarrhal and ulcerative stomatitis, mercurial stomatitis, and even, it is said, leukoplakia, are benefited by using it as a mouth-wash.

Ophthalmic surgeons are employing hydrogen peroxide in infective diseases of the conjunctiva and lachrymal apparatus. Membranous conjunctivitis, like membranous sore-throat, yields quickly to its influence. On the other hand, it is less useful than protargol in streptococcal and gonococcal conjunctivitis. A note of warning regarding its employment in ophthalmic work is sounded by Seitz, who reports that, after applying the dilute solution for a prolonged period to the healthy eye of a rabbit, corneal opacity was produced.

Skin ulcers of all kinds are refreshed, cleansed, and stimulated to cicatrise by hydrogen peroxide, and it has been reported that indolent ulcers of a tubercular nature have been healed when treated with $H_2O_2$ followed by iodoform. Foul cancerous ulcers and gangrenous sores are rendered clean and odourless when regularly bathed with the solution. On the intact skin hydrogen peroxide has no action whatever, nor is froth or foam produced. But it should be remembered that $H_2O_2$ is a powerful bleaching agent, and we should not therefore permit it to remain for any length of time in contact with the hair, especially if black or dark brown in colour.

### Peroxide in Diagnosis.

As a diagnostic agent, revealing the presence of pus, hydrogen peroxide has proved of much value. If the antrum of Highmore, *e.g.*, is suspected to contain pus, the puncture of its nasal wall by means of a fine trochar the cannula, followed by the injection of some of the solution, will soon settle the point, for if pus is present the foaming liquid at once comes pouring out of the nose, bearing on its surface characteristic yellow streaks. In suspected corneal ulcer, also, hydrogen peroxide is said to be as valuable as fluorescine, effervescence taking place at the spot where the surface is abraded.

Finally, let us note that the remedy, whether the official solution be employed in full strength or diluted, is inexpensive, easy to handle, and free from caustic or poisonous properties. In the pure form it sometimes gives rise to some smarting of the edges of a wound. When diluting the liquid care should be exercised to use only distilled water. As one of the drawbacks of hydrogen peroxide is its liability to undergo decomposition, a freshly made solution should always be used.

